# Evolutionary origins of Fibonacci phyllotaxis in land plants

**DOI:** 10.1016/j.heliyon.2024.e27812

**Published:** 2024-03-13

**Authors:** Takuya Okabe

**Affiliations:** Graduate School of Integrated Science and Technology, Shizuoka University, 3-5-1 Johoku, Hamamatsu, 432-8561, Japan

**Keywords:** Size-structure correlation, Heteroblasty, Plant morphology, Plant anatomy, Phyllotaxis transition

## Abstract

Fibonacci phyllotaxis is commonly seen in all major groups of land plants. While a precise correlation is found between the internal pattern of the primary vascular system and the external pattern of appendages on the stem surface, it remains a big question how this regularity of Fibonacci phyllotaxis came into being in the course of evolution. Here I address this problem with a model describing phylogenetic and ontogenetic changes in vascular phyllotaxis based on two hypotheses. The first is that the Fibonacci pattern of vascular connection is uniquely determined by the primary arrangement of incipient primordia, the sources of the primary signal system in vascular tissue differentiation. The second is that the surface-area-to-volume ratio of primary vascular tissues serves as a measure of fitness in evolution. The model explains the empirical rule on the manner in which vascular connection is reconfigured during ontogeny, especially during juvenile development. Fossil and phylogenetic evidence suggests that Fibonacci phyllotaxis appeared shortly after the innovation of indefinite lateral organ initiation in a regular sequence.

## 1 Introduction

Leaves of land plants are arranged in common patterns of Fibonacci phyllotaxis, despite their astonishing diversity in form, function and habit [1]. While the evolutionary origin of Fibonacci phyllotaxis remains one of the biggest unsolved problems in land plant evolution, it appears promising to consider it related to the evolution of the vascular system, for the external pattern of lateral organs (phyllotaxis, in the original sense of the term [1]) is strongly correlated with the internal architecture of the primary vascular system (vascular phyllotaxis [2], [3], [4], [5], [6], [7]). Morphological changes in early land plants have been addressed from two complementary perspectives, i.e., adaptive and non-adaptive [8]. On one hand, Bower's hypothesis is the most well-known adaptive approach to the evolution of primary vascular architecture [9], [10]. The primary vascular system serves as the supply system for the necessary nutrients and photosynthate to support the growth and development of the plant organism. Accordingly, the surface-to-bulk ratio of vascular tissues is considered important in exerting evolutionary pressure, driving natural selection: The higher surface-to-bulk ratios have the higher fitness. The correlation between increasing xylem complexity and body size is understood from this perspective. The size-structure correlation was first thoroughly investigated in pteridophytes [11]. In fern allies like *Psilotum* and *Lycopodium*, the correlation is reflected as changes in the configuration of the xylem. In ferns, even the type of stele may change markedly, from protostele through solenostele to dictyostele, as the stem thickness undergoes a progressive increase during growth [10], [12]. Bower argued for a similar pattern in the change of the primary xylem configuration during the course of evolution as a homoplastic adaptation (see ref. [13] for references and criticism). On the other hand, an alternative, non-adaptive view focuses on the passive consequence of change in factors controlling developmental activities, rather than a direct product of adaptive selection [14]. Mature vascular architecture is the consequence of specific patterns of development, growth and differentiation. The type of cell or tissue to differentiate, including primary vascular tissues, is under the controlling influence of some hormone like auxin, which is synthesized in the shoot apex and/or primordia of lateral appendages [8], [15], [16], [17]. Accordingly, vascular architecture changes in tandem with size, arrangement and complexity of lateral branch systems, the available hormone sources. Phylogenetic changes in stelar morphology are explained as a passive result of changes in growth and development [18], [19].

**Figure 1:**
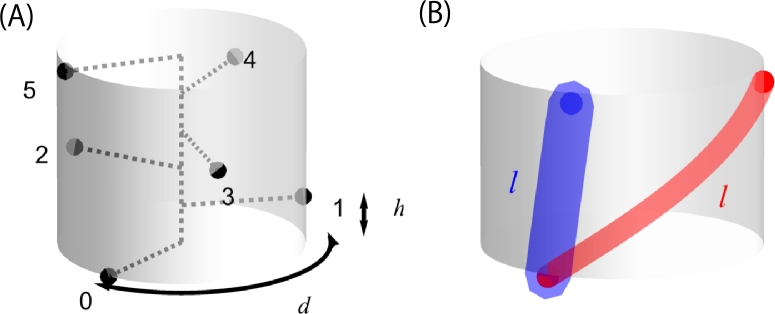
Cylindrical representation of helical phyllotaxis. (A) Leaf arrangement is represented in terms of divergence *d* and rise *h*. Leaves are shown as numbered points on a cylinder surface. (B) Nearby leaves are connected with a tract, whose surface-area-to-volume ratio (SA:V) is different for different patterns (thick blue versus thin red). Bower's hypothesis posits that the larger SA:V the more functional. For a fixed volume, SA:V increases as the length increases. Accordingly, a helical pattern with long tracts is favored for the benefit of the surrounding tissue.

Adopting the combination of the above two views, the present study focuses on the minute difference in the bulk-to-surface ratio of the vascular system between different vascular patterns (i.e., long or short in Fig. 1B). The most important contribution to the pattern dependence originates from the difference in length of the segments that interconnect leaves (or leaf traces). In Fibonacci phyllotaxis, the manner in which leaves are connected (vascular phyllotaxis) is not genetically determined but varies depending on growth conditions [20]. This variation is due to change in size of primordia relative to the shoot apical meristem [21]. Thus, it appears reasonable to hypothesize that there is a causal one-to-one relationship between the primary phyllotaxis and the vascular phyllotaxis. Indeed, a general picture of vascular differentiation in seed plants is that (1) phloem appears in a leaf trace, (2) phloem differentiates toward the leaf (acropetally), (3) xylem arises near the base and differentiates bidirectionally, acropetally in the leaf, basipetally in the stem [4]. In *Coleus blumei*, protoxylem differentiates bidirectionally from two sites, below a leaf primordium and in the stem, which are eventually connected through to the mature xylem in the stem to form a functional conduit. When the differentiating segments fail to meet each other, they are bridged laterally by differentiation of intervening protoxylem cells. Protoxylem differentiation may not follow a fixed pattern but vary leaf by leaf, reflecting non-uniformity in auxin concentration [22]. Thus, it is reasonable to consider that the positioning of leaf traces as entities (primary phyllotaxis) determines the patterning of procambial strands (vascular phyllotaxis), not vice versa. In contrast to decussate phyllotaxis, as in *Coleus*, the vascular arrangement in helical (spiral) phyllotaxis poses a peculiar problem due to the occurrence of various Fibonacci patternings, i.e., 1/2, 1/3, 2/5, 3/8, etc. As mentioned above, the Fibonacci patterning varies according to growth conditions [20], [21]. To discuss the potential adaptive value of this special characteristic, it is imperative to make a comprehensive comparison of all possible patterns, without begging the question of the existence of Fibonacci patterns. This task is, so to speak, a theoretical exploration through a morphospace of helical phyllotaxis [23], [24], [25]. It is shown that phylogenetic and ontogenetic changes in helical phyllotaxis are put in the same context of functionality as the size-structure correlation in the stem.

## 2 Model and results

**Figure 2:**
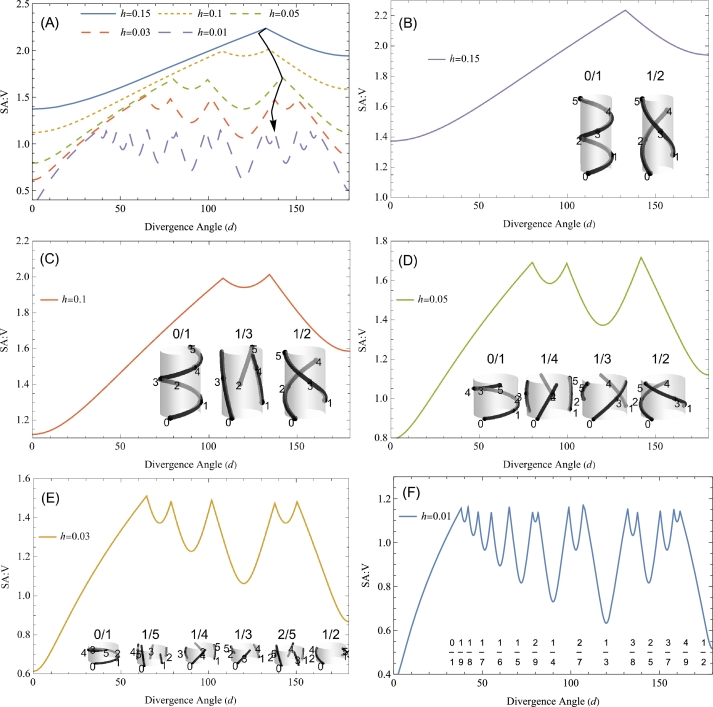
The surface-area-to-volume ratio (SA:V) is plotted against the primary divergence angle *d* (in degrees). (A) The following five cases for rise *h* = 0.01, 0.03, 0.05, 0.1 and 0.15 are shown altogether. A vertical arrow that connects peaks shows a trajectory to the 137.5^∘^ divergence. (B) Case *h* = 0.15. The inset shows two patterns, 0/1 and 1/2, to which the curves on the left and right of a peak correspond, respectively. (C) Case *h* = 0.1. Three segments correspond to three patterns in the inset, 0/1, 1/3 and 1/2. (D) Case *h* = 0.05. Four segments correspond to four patterns in the inset, 0/1, 1/4, 1/3 and 1/2. (E) Case *h* = 0.03. Six segments correspond to six patterns in the inset, 0/1, 1/5, 1/4, 1/3, 2/5 and 1/2. (F) Case *h* = 0.01. Fifteen segments correspond to fifteen patterns, 0/1, 1/9, 1/8, 1/7, 1/6, 1/5, 2/9, 1/4, 2/7, 1/3, 3/8, 2/5, 3/7, 4/9 and 1/2. A shallow kink at 108^∘^ is due to a 3/10 pattern about to emerge (360 × 3/10 = 108).

The primary arrangement of leaves is represented in terms of divergence *d* and rise *h*, such that the *n*-th leaf is positioned at $\left(\varphi_{n},z_{n})=(nd,nh\right)$ [26], [27], [28] (Fig. 1A). The rise, or the internodal distance, *h* reflects the size of the apical meristem relative to the leaf primordia. The larger the apical meristem, the larger *h*, for the primordium size being relatively constant (*h* is related to the plastochron ratio *a* in the centric representation [27], [29]). In the pattern of a phyllotactic fraction p/q, every leaf *n* is connected with leaf n+q to make a total of *q* vascular strands (passing through leaves 0, 1, 2, ⋯, q−1). Thus, the fraction (0/1, 1/2, etc.) designates the manner in which leaves are connected with each other (vascular phyllotaxis). The denominator is the number of bundles. Unlike the original concept of orthostichy [1], the numerator is not the number of turns between two consecutive leaves on the same bundle (which is not always a whole number or integer) but rather the nearest integer to it. Accordingly, the fractional representation does not imply that leaves are aligned parallel to the axis. The fraction for vascular phyllotaxis should not be confused with the primary divergence *d* for primary phyllotaxis. The present model posits that the fraction p/q is determined to minimize the segment length *l* between two adjacent leaves. The surface-area-to-volume ratio (SA:V) of this segment is given by $2\pi rl/(\pi r^{2}l)=2\sqrt{\pi l/v}$, assuming cylindrical form with radius *r*. The volume $v=\pi r^{2}l$ is factored out, because comparison is made for a fixed volume. The reason is that this is a problem of effective utilization of a given amount of resources. Bower focused attention on the change in surface area due to complication in surface shape of a fixed volume of vascular tissue [9]. This study focuses on the change in surface area of the vascular segment due to change in distance between adjacent leaves. Thus, the difference arises from differences in length *l* (Fig. 1B). The SA:V ratio increases as the tract segment elongates. Accordingly, a helical pattern with long tracts is favored for the benefit of the surrounding tissue. Fig. 2 shows the results for SA:V$=2\sqrt{\pi l}$ evaluated for various combinations of *d* and *h*.

When rise *h* is sufficiently large (h>0.29), all leaves are threaded through by a single bundle, i.e., a 0/1 phyllotaxis. As rise *h* decreases (h<0.29), the second pattern, a 1/2 phyllotaxis, comes into play. The SA:V takes a peak value around $d=130^{\circ }$ (Fig. 2B). The inset of Fig. 2B shows the two patterns 0/1 and 1/2 around the peak angle ($d=133^{\circ }$). The former (latter) is advantageous for a large (small) angle in terms of SA:V. As *h* decreases further (h<0.12), the third pattern, 1/3, creates a dent in the SA:V curve at $120^{\circ }$ (Fig. 2C). Next comes 1/4 (Fig. 2D) and so forth (Fig. 2EF). As a new pattern arises, optimal divergence at the previous stage (a local maximum, Fig. 2B) becomes non-optimal (a local minimum, Fig. 2C). Thus, optimal angle approaches a goal angle 137^∘^ as *h* decreases (an arrow in Fig. 2A) and Fibonacci patterns 1/2, 1/3, 2/5, etc. are brought about as most adaptive patterns.

**Figure 3:**
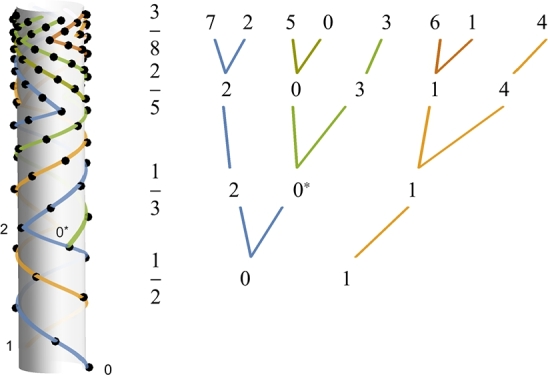
Sequential changes in vascular phyllotaxis during development. As *h* varies from 0.17 (bottom) to 0.006 (top), vascular phyllotaxis makes transitions from 1/2 through 1/3 and 2/5 to 3/8. A tree diagram shows how vascular bundles split upon phyllotaxis transition. The rule is that lower bundles (e.g., 0, 1 and 2 of 2/5) split while the rest (3 and 4 of 2/5) remain intact (to form eight bundles of 3/8). ($h(n)=1/{\left(\sqrt{6}+n/10\right)}^{2}$, *d* = 138^∘^.)

Fig. 3 shows changes in pattern as *h* varies from 0.17 (bottom) to 0.006 (top) ($d=138^{\circ }$). As the pattern changes from 1/2 to 1/3, one of the two bundles of the 1/2 pattern splits into two to form three bundles of 1/3. Specifically, bundle 0 of 1/2 splits into $0^{⁎}$ and 2 of 1/3, while bundle 1 remains undivided. In a similar manner, bundle $0^{⁎}$ of 1/3 splits into bundles 0 and 3 of 2/5, while bundle 1 of 1/3 splits into 1 and 4 of 2/5. This splitting pattern is represented by numbers to the right of the fractions (Fig. 3).

**Figure 4:**
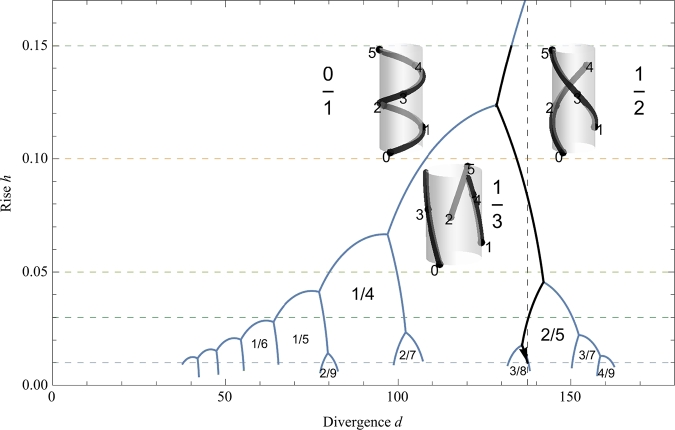
Morphospace of vascular phyllotaxis. Theoretically possible phyllotactic patterns are represented in the morphospace of primary divergence *d* and rise *h*. Each region represents a specific pattern designated with a phyllotactic fraction. As three inset images show, it is irrelevant whether vascular strands run parallel to the axis. Five horizontal lines (dashed) correspond to the five cases in Fig. 2B-F. A vertical line (dashed) is a 137.5 degrees angle. A zigzag trajectory (down solid arrow) corresponds to that in Fig. 2A. Narrow regions that emerge endlessly for small values of *h* are omitted for the sake of simplicity. Most plants occupy only the restricted regions of Fibonacci fractions 1/2, 1/3, 2/5, 3/8 and so forth.

Fig. 4 is the morphospace diagram in which each region represents a distinct pattern of vascular phyllotaxis. The five evolutionary stages in Fig. 2B-F correspond to five horizontal lines (dashed), while the developmental change in Fig. 3 follows a vertical dashed line.

## 3 Discussion

The significance of this study lies in providing hypothetical evolutionary changes in helical phyllotaxis based on the Bower hypothesis for changes in stem structure. It is generally found that a progressive increase in phyllotactic complexity accompanies an increase in size of the axis [12]. Therefore, a plausible scenario is that the evolution proceeds from simple to complex, as seen in the development. In the present model, this process is represented as a gradual decrease in *h*. Fibonacci phyllotaxis with the primary divergence of 137.5 degrees evolves from gradual progression through lower to higher phyllotaxis order (an arrow in Fig. 2A and in Fig. 4) under the hypothesis that SA:V serves as a measure of reproductive fitness [9]. The evolutionary stage may be referred to by the highest fraction attained in that stage. In the stage 1/2, evolutionary pressure causes the primary divergence towards around 130 degrees (Fig. 2B). In the next stage 1/3, the optimal angle splits into about 110 degrees and 140 degrees (Fig. 2C). The latter leads to the 137.5^∘^ angle, while the former splits into 80 and 100 degrees (Fig. 2D). Thus, the early stages up to the stage 2/5 (Fig. 2E) are of importance for understanding the current predominance of Fibonacci phyllotaxis.

Most vascular plants progress through phyllotaxis transitions before attaining maturity, especially during seedling/sporeling development [4], [5], [6], [12]. The strict regularity in the branching pattern of vasculature, as described in Fig. 3, has been long recognized [2], [30], [28]. The empirical rule is that, when phyllotaxis makes a transition from m/n to p/q (q>n), the first q−n bundles from among the *n* bundles of the m/n system split into two to form *q* bundles of p/q (i.e., n+(q−n)=q). The transition is caused by the change in size of the shoot apex relative to primordia [4], [26]. In the present model, this empirical rule follows from the assumptions that divergence angle is a fixed trait, and that an initiated leaf is passively connected to a nearby leaf below. The rationale behind the latter is that vascular phyllotaxis is determined by the geometric configuration of signal sources of vascular tissue differentiation [8], [19].

The cylindrical representation in phyllotaxis was first introduced by Bravais and Bravais to analyze what set of parastichy numbers are seen for a given value of divergence angle [31]. Since then, there have been a plethora of theoretical arguments based on this theoretical framework (see Jean [28] and references therein). Along with the centric representation [32] and others, the classical geometrical models in early studies were expounded by van Iterson [33] (see Erickson [34] for a concise review). Technically, the present study is an application of this line of analysis. However, the novel view from an adaptive perspective cannot be overemphasized. Plants in spiral/helical phyllotaxis change the phyllotactic pattern while they grow. Young seedlings exhibit a lower-order pattern whereas older shoots show a higher-order pattern. Schwendener put forward a mechanical mechanism for this transition based on the Dachstuhl (timber roof truss) model [35], which suggests change in divergence angle similar to the zigzag course in Fig. 2A (cf. Fig. 3.10 of ref. [34]). The chemical inhibitor mechanism for positioning leaf primordia follows this line of thought [26], [36]; auxin depletion may act as the inhibitory field around a primordium [37], [38], [39]. Thus, the existing models argue that transitions from a lower to higher-order patterns are caused in the process of growth and development of a shoot apex, i.e., in a developmental timescale. By contrast, the present study argues that this transition has occurred in an evolutionary timescale, so that the 137.5^∘^ Fibonacci angle was achieved as a convergent characteristic of land plants. It is important not to confuse these analyses at different levels, proximate and ultimate causes. Mechanisms at different levels have different implications. There is no point in arguing whether mechanisms at different levels are exclusive alternatives [40].

**Figure 5:**
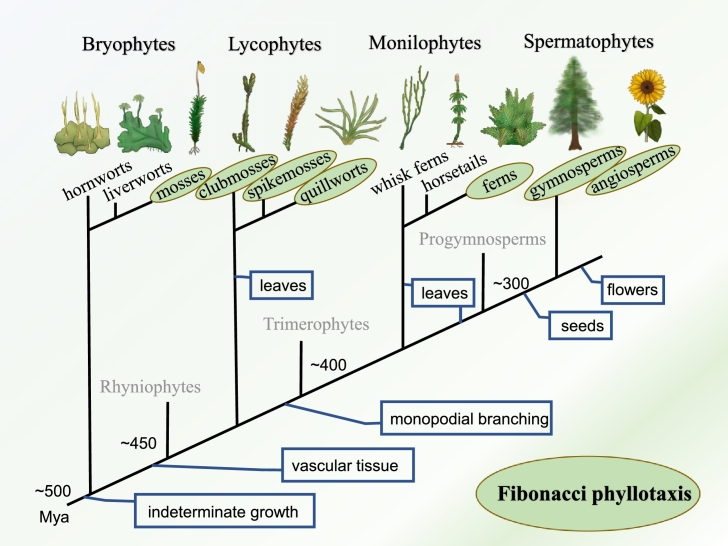
Land plant phylogeny highlighting the occurrence of Fibonacci phyllotaxis. Fibonacci phyllotaxis occurs in seed plants (Spermatophytes), ferns, clubmosses (*Huperzia*), spike mosses (*Selaginella*), quillworts (*Isoetes*) and peristomate mosses. It is not so common in lycophytes and ferns as in seed plants. Important evolutionary innovations and extinct groups are also shown on a phylogenetic tree (simplified after refs. [47], [48]). Mya: million years ago.

Fibonacci phyllotaxis occurs in all major clades of land plants (Fig. 5). In the progymnosperms of the Late Devonian, branch traces of *Callixylon* (main stems of *Archaeopteris*) are arranged in 2/5, 3/8 and 5/13 patterns [41]. The recent finding of non-Fibonacci phyllotaxis in an Early Devonian lycopod, *Asteroxylon mackiei*, has significant implications on the evolutionary origin of phyllotaxis [42]. Most importantly, it suggests that Fibonacci phyllotaxis in lycopods evolved lately in the Carboniferous [43], for similar non-Fibonacci patterns as in living species are found in Devonian and Early Carboniferous species [44], [45]. Since leaves evolved independently in lycophytes and euphyllophytes, Fibonacci phyllotaxis present in living members of both groups may have evolved independently. In bryophytes, Braun remarked that 3/8 and 5/13 are equally very common in mosses, citing plenty of species with peristome teeth (Bryidae, etc.) [1].

While non-Fibonacci patterns in helical phyllotaxis are generally rare, it is not rare to see non-helical patterns like distichy and decussate, in which leaves are arranged in straight lines parallel to the stem. Most phyllotactic patterns are so well-ordered that it appears unlikely that they have no evolutionary/functional significance. If they have some evolutionary significance, any mechanism that explains a functional advantage of helical patterns should fail to explain that of non-helical patterns. The present study argues that helicity is favored because long and thin xylem serves the surrounding living tissue better than short and thick one (Fig. 1B), if different patterns with the same volume of xylem are compared with each other. In real life, there may be constraints that could undermine this argument. For instance, there should be a lower limit of thickness to be a functional tube. If the xylem volume is a limiting factor, it also makes sense to shortening inter-leaf distances by arranging them non-helically on top of each other.

The adaptive perspective provides a neat explanation for the ubiquity of Fibonacci phyllotaxis as a necessary result of extending the vascular coverage during the course of evolution. Still, the same phyllotaxis on the shoot apical meristems of distantly related plants with significantly different structures is tremendously puzzling from a developmental viewpoint. As a matter of fact, whether Fibonacci or not is not a matter of life or death to an individual plant. Far more important for plants' survival is the innovation of mechanisms for arranging organs not only in lateral position (away from shoot tips) but in a regular sequence, i.e., refinement and stabilization of indeterminate growth [46]. A comparative analysis between different lineages would show to what extent these mechanisms are homologous by descent.

## Additional information

No additional information is available for this paper.

## Ethics declaration

Review and/or approval by an ethics committee was not needed for this study because this study does not include any animal and human research.

## CRediT authorship contribution statement

**Takuya Okabe:** Writing – review & editing, Writing – original draft, Visualization, Validation, Supervision, Software, Resources, Project administration, Methodology, Investigation, Funding acquisition, Formal analysis, Conceptualization.

## Declaration of Competing Interest

The author declares the following financial interests/personal relationships which may be considered as potential competing interests: Takuya Okabe reports financial support was provided by 10.13039/501100001691Japan Society for the Promotion of Science (no. 21K12047).

## Data Availability

No data was used for the research described in the article.
